# Protective Activity of Aspirin Eugenol Ester on Paraquat-Induced Cell Damage in SH-SY5Y Cells

**DOI:** 10.1155/2021/6697872

**Published:** 2021-08-04

**Authors:** Zhen-Dong Zhang, Ya-Jun Yang, Zhe Qin, Xi-Wang Liu, Shi-Hong Li, Li-Xia Bai, Jian-Yong Li

**Affiliations:** Key Lab of New Animal Drug Project of Gansu Province, Key Lab of Veterinary Pharmaceutical Development of Ministry of Agriculture and Rural Affairs, Lanzhou Institute of Husbandry and Pharmaceutical Sciences of CAAS, Lanzhou 730050, China

## Abstract

Aspirin eugenol ester (AEE) is a new pharmaceutical compound esterified by aspirin and eugenol, which has anti-inflammatory, antioxidant, and other pharmacological activities. The aim of this study was to investigate the protective effect of AEE on paraquat- (PQ-) induced cell damage of SH-SY5Y human neuroblastoma cells and its potential molecular mechanism. There was no significant change in cell viability when AEE was used alone. PQ treatment reduced cell viability in a concentration-dependent manner. However, AEE reduced the PQ-induced loss of cell viability. Flow cytometry, terminal deoxynucleotidyl transferase dUTP nick end labeling (TUNEL), and 4′6-diamidino-2-phenylindole (DAPI) staining were used to evaluate cell apoptosis. Compared with the PQ group, AEE pretreatment could significantly inhibit PQ-induced cell damage. AEE pretreatment could reduce the cell damage of SH-SY5Y cells induced by PQ via reducing superoxide anion, intracellular reactive oxygen species (ROS), and mitochondrial ROS (mtROS) and increasing the levels of mitochondrial membrane potential (ΔΨ*m*). At the same time, AEE could increase the activity of glutathione peroxidase (GSH-Px), catalase (CAT), and superoxide dismutase (SOD) and decrease the activity of malondialdehyde (MDA). The results showed that compared with the control group, the expression of p-PI3K, p-Akt, and Bcl-2 was significantly decreased, while the expression of caspase-3 and Bax was significantly increased in the PQ group. In the AEE group, AEE pretreatment could upregulate the expression of p-PI3K, p-Akt, and Bcl-2 and downregulate the expression of caspase-3 and Bax in SH-SY5Y cells. PI3K inhibitor LY294002 and the silencing of PI3K by shRNA could weaken the protective effect of AEE on PQ-induced SH-SY5Y cells. Therefore, AEE has a protective effect on PQ-induced SH-SY5Y cells by regulating the PI3K/Akt signal pathway to inhibit oxidative stress.

## 1. Introduction

Parkinson's disease (PD) is a typical age-related chronic progressive disease, and it is also one of the most common neurodegenerative diseases [1]. PD primarily results from the progressive degeneration of dopaminergic neurons in the substantia nigra pars compacta (SNpc) [2]. Clinically, PD is associated with motor impairments including bradykinesia, rigidity, resting tremor, and gait disturbance [3]. However, the mechanism remains elusive. Studies showed that oxidative stress, impairment of mitochondrial, and apoptotic cascade activation play important roles in the occurrence and development of PD [4, 5]. Similarly to other neurodegenerative diseases, PD patients display increased levels of oxidative stress and ROS, decreased mitochondrial membrane potential, and activated caspase cascade [6, 7].

Although the causes of PD are still unclear, the evidence strongly suggests that mitochondrial dysfunction and oxidative stress are involved in its pathogenesis [8]. Factors that increase the risk of PD have been identified, including increased age, exposure to environmental toxins, and genetic factors [9, 10]. These factors affect the function of the mitochondria through oxidative stress and then lead to neuronal apoptosis [11]. Among them, environmental factors are considered to play the key role in neurotoxicity, especially factors leading to oxidative stress, such as pesticides or heavy metals [12]. PQ is a highly toxic nonselective herbicide that is widely used throughout the world [13–15]. Epidemiological studies showed that acute PQ poisoning can lead to severe brain damage and increase the incidence of PD [16]. Some studies showed that long-term low-dose exposure to PQ could induce the formation of *α*-synuclein aggregates, which could change the catabolism of dopamine and inactivate tyrosine hydroxylase [17, 18].

There is still lack of neuroprotective drug for treatment of neurodegenerative diseases; some drugs are used to attenuate symptoms [19–22]. Aspirin provided a clear neuroprotection against tetrahydropyridine toxicity on the striatal and nigral levels in murine Parkinson's model [19]. Aspirin had a protective effect on nerve injury induced by 1-methyl-4-phenylpyridiniumion and 6-hydroxydopamine in rats [22]. The combined use of docosahexaenoic acid and aspirin could significantly promote the expression of neurotrophic factors and promote the formation of PPAR*α* and RXR*α* heterodimer, which provides a new method for the treatment of PD [21]. NOSH-aspirin, a novel nitric oxide and hydrogen sulfide-releasing hybrid, attenuates neuroinflammation induced by microglial and astrocytic activation [20]. Similarly, eugenol also has a neuroprotective effect [23, 24]. The main component of grassleaf sweetflag rhizome is eugenol, which could cross the blood-brain barrier and protect neurons. Studies have shown that eugenol has a neuroprotective effect on PC12 cells induced by amyloid-beta42 [24]. Behavioral and biochemical results showed the neuroprotective effects of eugenol and isoeugenol on acrylamide-induced neuropathy in rats [23].

As a new compound, AEE plays an active role in many aspects [25–34]. AEE has not only the effects of anti-inflammation, antithrombosis, and antiblood stasis but also the effect of antiatherosclerosis and other cardiovascular diseases. It is not clear whether AEE can play a neuroprotective role in neurodegenerative diseases. The purpose of this study was to explore whether AEE can attenuate PQ-induced oxidative damage in SH-SY5Y cells and its possible mechanism.

## 2. Materials and Methods

### 2.1. Chemicals

Aspirin eugenol ester (purity 99.5%) was prepared in Lanzhou Institute of Husbandry and Pharmaceutical Sciences of CAAS (Lanzhou, China). Dimethyl sulfoxide was supplied by Sigma (St. Louis, MO). Methyl viologen dichloride was purchased from Aladdin (Shanghai, China). Dulbecco's modified Eagle medium and fetal bovine serum were from Gibco (Grand Island, NY, USA). One Step TUNEL apoptosis assay kit, puromycin dihydrochloride, bicinchoninic acid assay kit, glutathione peroxidase kit, catalase assay kit, DAPI staining solution, and DAF-FM diacetate kit were obtained from Beyotime (Shanghai, China). Anti-PI3K, anti-Akt, anti-phosphorylation-PI3K (Tyr458), and anti-phosphorylation-Akt were purchased from Cell Signaling Technology, Inc. (Beverly, MA, USA). Anti-Bax, anti-Bcl-2, and anti-caspase-3 were from Abcam (Cambridge, MA, USA). An Annexin V/FITC apoptosis detection kit was from BD Biosciences (San Diego, CA, USA). PI3-kinase inhibitor LY294002 was purchased from MedChemExpress LLC (New Jersey, USA). Lipofectamine™ 3000 transfection reagent was purchased from Thermo Fisher (Invitrogen, USA). Lentivirus control and PI3K shRNA (U6-MCS-Ubiquitin Cherry-IRES-puromycin) were purchased from GeneChem (Shanghai, China).

### 2.2. Cell Cultures and Cell Treatment

SH-SY5Y human neuroblastoma cells were routinely maintained in 10% fetal bovine serum (FBS), 1% GlutaMAX, 1% sodium pyruvate, 1% nonessential amino acids (NEAA), 87% 1 : 1 mix of F12, and modified Eagle medium (MEM) media supplemented with 2 mM L-glutamine at 37°C under humidified atmospheric conditions containing 5% CO_2_. Media were replaced every two days. Subcultures were performed with the trypsin-EDTA method. Experiments were subsequently conducted on 6-7 passages of cells. SH-SY5Y human neuroblastoma cells were randomly divided into three groups (*n* = 6): control group, PQ group, and AEE pretreatment group. Cells in the control group were incubated with the normal growth conditions. Those in the PQ group were incubated with the medium containing 250 *μ*M of PQ for 24 h. In the AEE pretreatment groups, the cells were cultured with the medium containing different concentrations of AEE (1, 2.0, and 4.0 *μ*M) for 24 h before they were incubated with medium containing 250 *μ*M PQ for 24 h.

### 2.3. Cell Viability

The viability of SH-SY5Y cells was detected by using a cell counting kit-8 (CCK-8) following the instructions of the manufacturer [28].

### 2.4. Flow Cytometric Analysis

SH-SY5Y cells (1 × 10^5^/well) were seeded in 6-well plates. After treatments, cells were assessed using the Annexin V/FITC apoptosis detection kit according to the manufacturer's protocols [28]. The cells were sorted by a flow cytometer (BD FACSVerse, CA, USA), and the data were analyzed with FlowJo 7.6. For proper statistical analysis, more than 10000 cells per group were counted, and each assessment was repeated three times.

### 2.5. Measurement of Mitochondrial Membrane Potential (ΔΨ*m*)

The ΔΨ*m* was determined using MitoTracker® Red CMXROS (Invitrogen; Thermo Fisher Scientific, Inc.). Briefly, the cells (1 × 10^4^/well) were seeded in 12-well plates. MitoTracker® Red probe was directly added into the culture media and incubated for 30 min at 37°C in the dark. Images were captured using a scanning laser confocal microscope (LSM800; Carl Zeiss, Germany).

### 2.6. Measurement of Intracellular and Mitochondrial Reactive Oxygen Species (ROS) and Superoxide Anion

Intracellular and mitochondrial ROS generation, and superoxide anion were measured using a DCFH-DA or MitoSOX™ red probe or dihydroethidium (DHE) as described previously method [35].

### 2.7. DAPI and TUNEL Staining

SH-SY5Y cells (1 × 10^4^/well) were seeded into 12-well culture plates. After treatment, cells were washed with PBS and fixed with 4% paraformaldehyde in PBS at 25°C for 30 min. After the cells were washed with PBS twice, 0.3% Triton X-100 PBS was added and incubated at 25°C for 5 min. TUNEL detection solution was added. After incubation of cells at 37°C for 1 h, DAPI staining solution was added and incubated at room temperature for 20 min. The cells were washed with PBS. Images were captured using a scanning laser confocal microscope (LSM800, Carl Zeiss, Germany). The TUNEL index (%) was based on the fluorescence microscopy detection of TUNEL-positive cells and DAPI-positive cells.

### 2.8. Determination of MDA, SOD, GSH-Px, and CAT in SH-SY5Y Cells

The activities of MDA, SOD, GSH-Px, and CAT in SH-SY5Y cells were assessed using the corresponding commercial kits according to the manufacturer's protocols [36, 37].

### 2.9. Protein Expression Analysis

The expressions of Bcl-2 (1 : 1000), Bax (1 : 5000), caspase-3 (1 : 500), PI3K (1 : 1000), Akt (1 : 1000), phospho-PI3K (1 : 1000), and phospho-Akt (1 : 1000) were assessed by western blot analysis. Protein was extracted from SH-SY5Y cells with RIPA lysis buffer containing 1 mM PMSF and a cocktail of protease and phosphatase inhibitors. The protein concentration was quantified using a bicinchoninic acid (BCA) assay kit. All measured proteins were normalized to *β*-actin level, and later, p-protein/total protein was calculated. Protein samples were separated by SDS-PAGE using 4-20% precast gradient polyacrylamide gels (Shanghai Suolaibao Bio-Technology Co., Ltd., Shanghai, China). After separation by SDS-PAGE, proteins were transferred to a PVDF membrane. The results were detected using G:BOX Chemi XRQ imaging system (Cambridge, Britain). ImageJ software (NIH) was used for quantification of band intensities, and intensities were normalized with total protein or the load control. Three duplicate wells were set in each group for the assay.

### 2.10. Cell Transfection

Lentiviral vectors expressing PI3K shRNA or control shRNA were obtained from GeneChem (Shanghai, China). Following the manufacturer's protocol, SH-SY5Y cells were cotransfected with lentivirus and packaging vectors using Lipofectamine 3000. Lentiviruses were harvested 48 h after transfection, centrifuged, and filtered through 0.45 *μ*m membrane filters (Millipore). Lentiviruses were transduced in 50% confluent SH-SY5Y cells. Stable clones were selected for 1 week by using puromycin (1 *μ*g/mL).

### 2.11. Determination of Apoptosis after Inhibition of Signal Pathway

To further examine the role of the PI3K/Akt signal pathway in AEE inhibiting PQ-induced apoptosis in SH-SY5Y cells, the PI3K signaling pathway in the SH-SY5Y cells was inhibited by shRNA and inhibitor LY 294002 against PI3K. In this part, it was divided into eleven groups. These SH-SY5Y cells were treated with 4.0 *μ*M AEE and PQ according to the protocol described in Section 2.2.

### 2.12. Statistical Analysis

Statistical analysis was carried out using the SAS 9.2 (SAS Institute Inc., NC, USA). All data are presented as the means ± SD. The differences among different treatment groups were analyzed with one-way ANOVA followed with Duncan's multiple comparisons. Statistical significance was considered at *p* < 0.05. All experiments were performed as three independent replicates.

## 3. Results

### 3.1. AEE Protects the Cell Viability of PQ-Stimulated SH-SY5Y Cells

**Figure 1:**
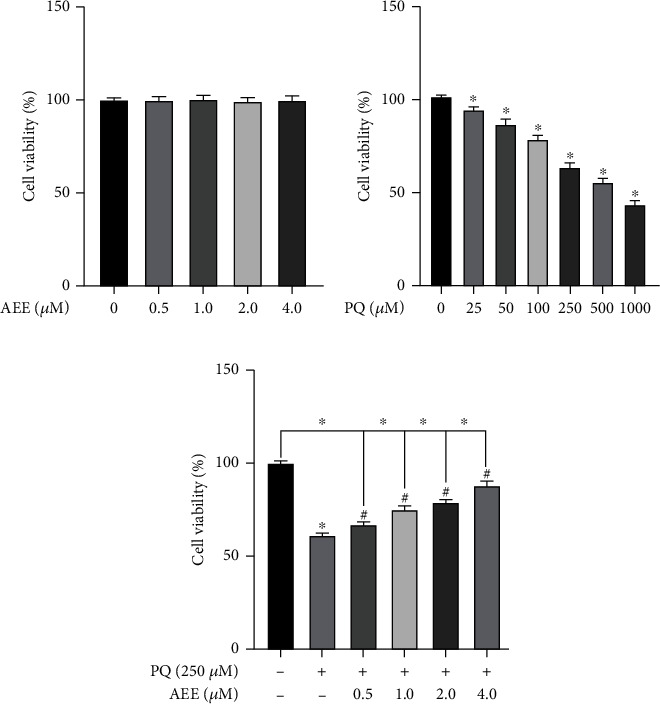
Protective effects of AEE on the cell viability of PQ-induced SH-SY5Y cells. (a) Different concentrations of AEE had no effect on the viability of SH-SY5Y cells. (b) PQ induced a concentration dependent decreased in SH-SY5Y cell viability. (c) AEE could significantly inhibit the decrease of SH-SY5Y cell viability induced by PQ. Values are presented as the means ± SD where applicable (*n* = 6). ^∗^*p* < 0.05 compared with the control group, ^#^*p* < 0.05 compared with the PQ group, and no significant difference (NS) *p* > 0.05. “+”: with the treatments in the SH-SY5Y cells; “−”: without the treatments in the SH-SY5Y cells.

AEE (0.5, 1, 2, and 4 *μ*M) alone had no significant effect on the viability of SH-SY5Y cells (Figure 1(a)). The SH-SY5Y cells were treated to various concentrations of PQ (25, 50, 100, 250, 500, and 1000 *μ*M/mL) for 24 h. The results showed that treatment with PQ reduced cell viability in a concentration-dependent manner. When the cells were treated with 250 *μ*M PQ for 24 h, the cell viability decreased significantly compared with the control group (Figure 1(b)). Therefore, the concentration of 250 *μ*M PQ was used in further experiments. Compared with the PQ group, the lower concentrations of AEE (0.5, 1, and 2 *μ*M) only partially improved viability, and the concentration of 4 *μ*M AEE was the most effective (Figure 1(c)).

### 3.2. AEE Inhibits PQ-Induced Apoptosis in SH-SY5Y Cells

**Figure 2:**
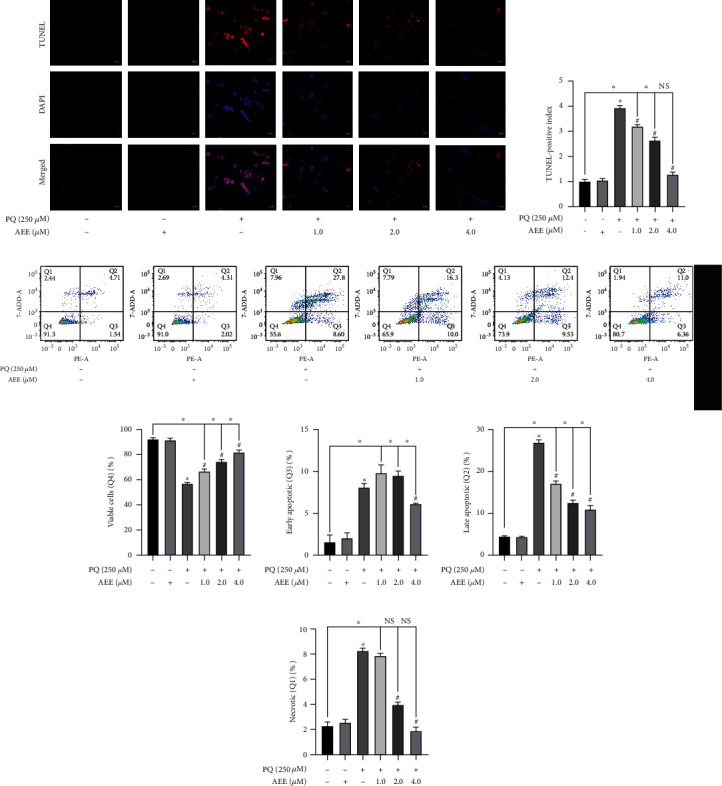
Effects of AEE on apoptosis in PQ-induced SH-SY5Y cells. (a, b) AEE significantly inhibited PQ-induced apoptosis of SY5Y cells by DAPI and TUNEL staining. Scale bar = 20 nm. Values are presented as the means ± SD where applicable (*n* = 6). (c–g) AEE significantly inhibited PQ-induced apoptosis of SH-SY5Y cells by flow cytometry. Values are presented as the means ± SD where applicable (*n* = 3). ^∗^*p* < 0.05 compared with the control group, ^#^*p* < 0.05 compared with the PQ group, and no significant difference (NS) *p* > 0.05. “+”: with the treatments in the SH-SY5Y cells; “−”: without the treatments in the SH-SY5Y cells.

The apoptosis morphology was evaluated by DAPI staining, and TUNEL assay was performed to assess apoptosis-induced DNA fragmentation. Compared with the control group, the number of TUNEL-positive cells (red fluorescence) in SH-SY5Y cells treated with PQ increased significantly. Compared with the control group, there was no significant difference in the number of TUNEL-positive cells (red fluorescence) in the 4.0 *μ*M AEE+PQ treatment group (Figures 2(a) and 2(b)). The results of Annexin V-PE/7-AAD flow cytometry showed that PQ could significantly inhibit the survival of SH-SY5Y cells, while AEE could reduce the apoptosis of SH-SY5Y cells induced by PQ (Figures 2(c) and 2(d)). In addition, PQ could increase early apoptosis (Q3), late apoptosis (Q2), and necrosis (Q1) of SH-SY5Y cells, while 4 *μ*M AEE could significantly attenuate the above phenomenon (Figures 2(e)–2(g)). The above results showed that AEE could inhibit the apoptosis of SH-SY5Y cells induced by PQ with an obvious concentration-dependent manner.

### 3.3. AEE Attenuates PQ-Induced Oxidative Stress in SH-SY5Y Cells

**Figure 3:**
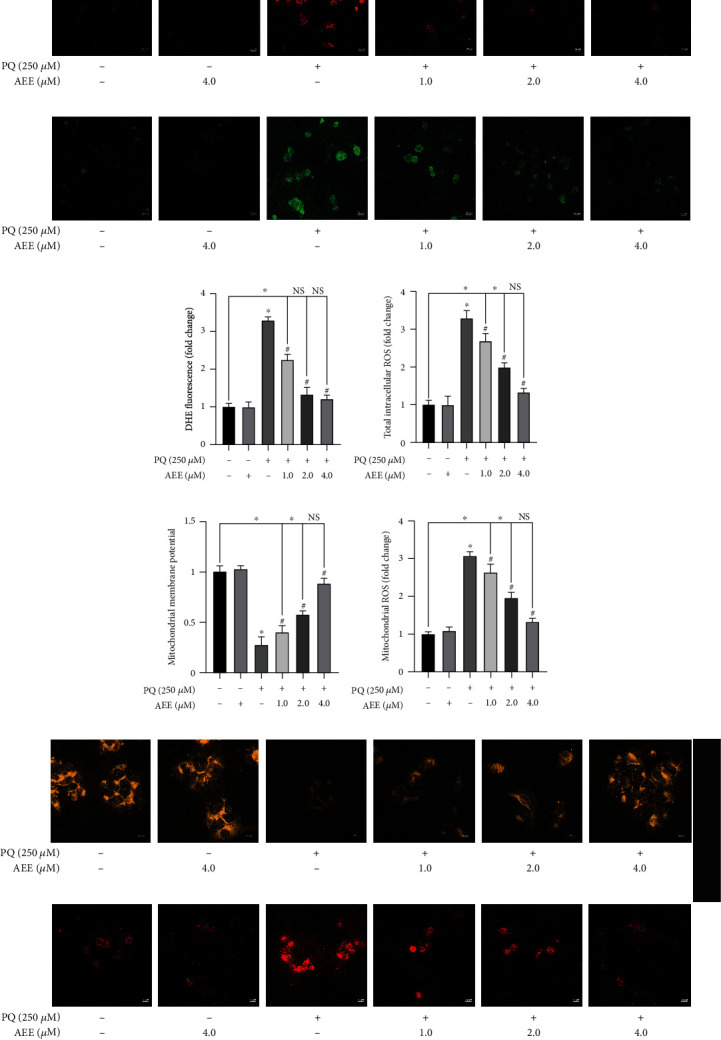
AEE antagonizes PQ-induced oxidative stress in SH-SY5Y cells. (a, c) AEE significantly inhibited the increase of superoxide anion level in SH-SY5Y cells induced by PQ. (b, d) AEE significantly inhibited the increase of ROS level in SH-SY5Y cells induced by PQ. (e, g) AEE significantly inhibited the decrease of mitochondrial membrane potential induced by PQ in SH-SY5Y cells. (f, h) AEE significantly inhibited the increase of mitochondrial ROS in SH-SY5Y cells induced by PQ. Values are presented as the means ± SD where applicable (*n* = 6). ^∗^*p* < 0.05 compared with the control group, ^#^*p* < 0.05 compared with the PQ group, and no significant difference (NS) *p* > 0.05. “+”: with the treatments in the SH-SY5Y cells; “−”: without the treatments in the SH-SY5Y cells.

To verify the changes of redox status of SH-SY5Y cells, the levels of superoxide anion, intracellular ROS, and mitochondrial reactive oxygen species (mtROS) were detected. Analysis involved determination of pixels assigned to each cell using ImageJ software. Three images in each group were analyzed and each image analyzed 6 cells. AEE alone did not change the levels of superoxide anion, intracellular ROS, and mtROS. Compared with the control group, PQ could significantly increase the levels of superoxide anion, intracellular ROS, and mtROS. However, (4.0 *μ*M) AEE pretreatment significantly inhibited PQ-induced superoxide anion, intracellular ROS, and mtROS levels in SH-SY5Y cells (Figures 3(a)–3(d), 3(f), and 3(h)). In addition, the mitochondrial membrane potential (ΔΨ*m*) was further detected. The results showed that AEE alone did not affect ΔΨ*m* of SH-SY5Y cells (Figures 3(e) and 3(g)), compared with the control group. AEE pretreatment could significantly increase ΔΨ*m* in SH-SY5Y cells, compared with the PQ group (Figures 3(e) and 3(g)). Compared with the control group, there was no significant difference in the 4.0 *μ*M AEE+PQ treatment group. The results showed that AEE could significantly alleviate the mitochondrial dysfunction of SH-SY5Y cells via inhibiting intracellular ROS, mtROS, and superoxide anion levels.

### 3.4. AEE Enhances the Activities of ROS Scavenging Enzymes in PQ-Stimulated SH-SY5Y Cells

**Figure 4:**
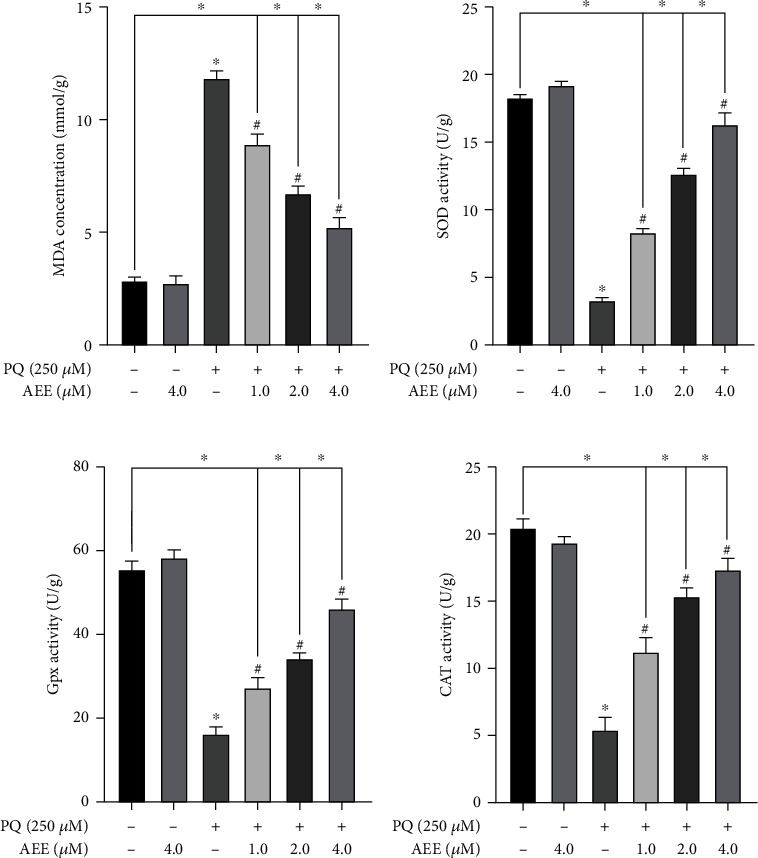
AEE enhances the activities of ROS scavenging enzymes in PQ-induced SH-SY5Y cells. (a) AEE significantly inhibited the increase of MDA level in SH-SY5Y cells induced by PQ. (b) AEE significantly inhibited the decrease of SOD level in SH-SY5Y cells induced by PQ. (c) AEE significantly inhibited the decrease of GSH-Px level in SH-SY5Y cells induced by PQ. (d) AEE significantly inhibited the decrease of CAT level in SH-SY5Y cells induced by PQ. Values are presented as the means ± SD where applicable (*n* = 6). ^∗^*p* < 0.05 compared with the control group, ^#^*p* < 0.05 compared with the PQ group, and no significant difference (NS) *p* > 0.05. “+”: with the treatments in the SH-SY5Y cells; “−”: without the treatments in the SH-SY5Y cells.

The activities of SOD, GSH-Px, CAT, and MDA were detected to explore the protective effect of AEE on SH-SY5Y cell induced by PQ. PQ could significantly increase the activity of MDA and decrease the activity of SOD, GSH-Px, and CAT, compared with the control group. However, AEE pretreatment could significantly increase the activities of SOD, GSH-Px, and CAT and decrease the activity of MDA (Figures 4(a)–4(d)). These results suggested that AEE pretreatment may attenuate PQ-induced oxidative damage in SH-SY5Y cells via increasing the activity of ROS scavenging enzymes and inhibiting the activity of MDA.

### 3.5. AEE Regulates the Expression of Apoptosis-Related Proteins in SH-SY5Y Cells Induced by PQ

**Figure 5:**
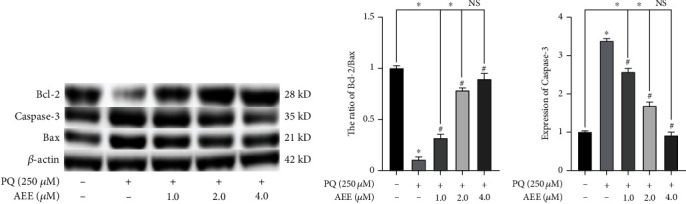
AEE regulates the expression of Bcl-2, Bax, and caspase-3 in SH-SY5Y cells induced by PQ. (a, b) AEE could significantly inhibit the decrease of Bcl-2/Bax ratio induced by PQ in SH-SY5Y cells. (a, c) AEE significantly inhibited the increase of caspase-3 protein in SH-SY5Y cells induced by PQ. Load in each lane was 40 *μ*g protein. Values are presented as the means ± SD where applicable (*n* = 3). ^∗^*p* < 0.05 compared with the control group, ^#^*p* < 0.05 compared with the PQ group, and no significant difference (NS) *p* > 0.05. “+”: with the treatments in the SH-SY5Y cells; “−”: without the treatments in the SH-SY5Y cells.

To further explore the molecular mechanism of AEE attenuating PQ-induced apoptosis in SH-SY5Y cells, the western blotting was used to detect the expression of apoptotic proteins (caspase-3, Bcl-2, and Bax). As shown in Figures 5(a)–5(d), compared with the control group, PQ could significantly inhibit the expression of Bcl-2 and significantly promote the expression of Bax and caspase-3. However, AEE pretreatment could significantly reverse the above changes. Compared with the control group, the ratio of Bcl-2/Bax and the expression of caspase-3 were not significantly different in the 4.0 *μ*M AEE+PQ treatment group.

### 3.6. AEE Regulates the PI3K/Akt Signaling Pathways in SH-SY5Y Cells

**Figure 6:**
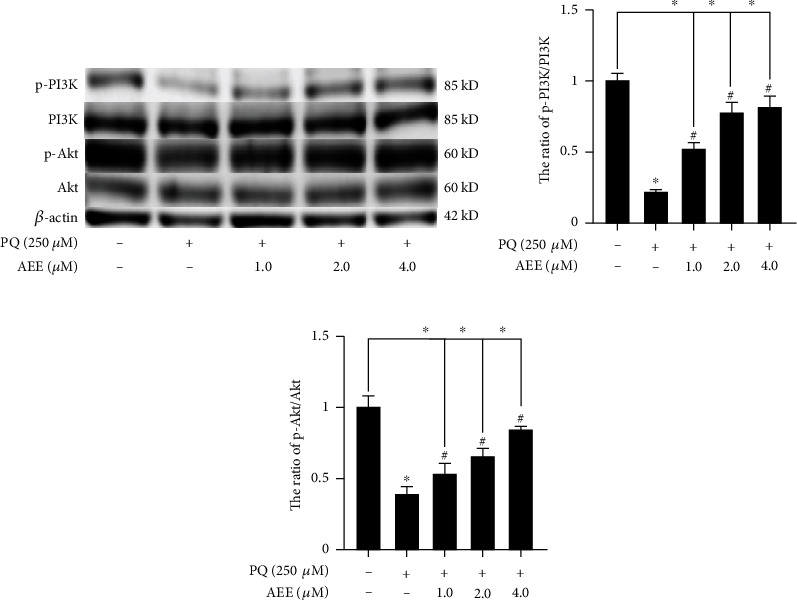
AEE regulates the expression of p-PI3K, PI3K, p-Akt, and Akt in SH-SY5Y cells induced by PQ. (a–c) AEE could increase the ratio of p-PI3K/PI3K and the ratio of p-AKT/Akt in SH-SY5Y cells induced by PQ. Values are presented as the means ± SD where applicable (*n* = 3). ^∗^*p* < 0.05 compared with the control group, ^#^*p* < 0.05 compared with the PQ group, and no significant difference (NS) *p* > 0.05. “+”: with the treatments in the SH-SY5Y cells; “−”: without the treatments in the SH-SY5Y cells.

Compared with the control group, the expression of p-Akt and p-PI3K in the PQ group decreased significantly. However, compared with the PQ group, AEE pretreatment could significantly upregulate the expression of p-Akt and p-PI3K in SH-SY5Y cells (Figures 6(a) and 6(b)). AEE pretreatment had no significant effect on the expression of Akt and PI3K in SH-SY5Y cells induced by PQ. These results suggested that AEE may possess protective potentials on PQ-induced SH-SY5Y cells via the PI3K/Akt pathway.

**Figure 7:**
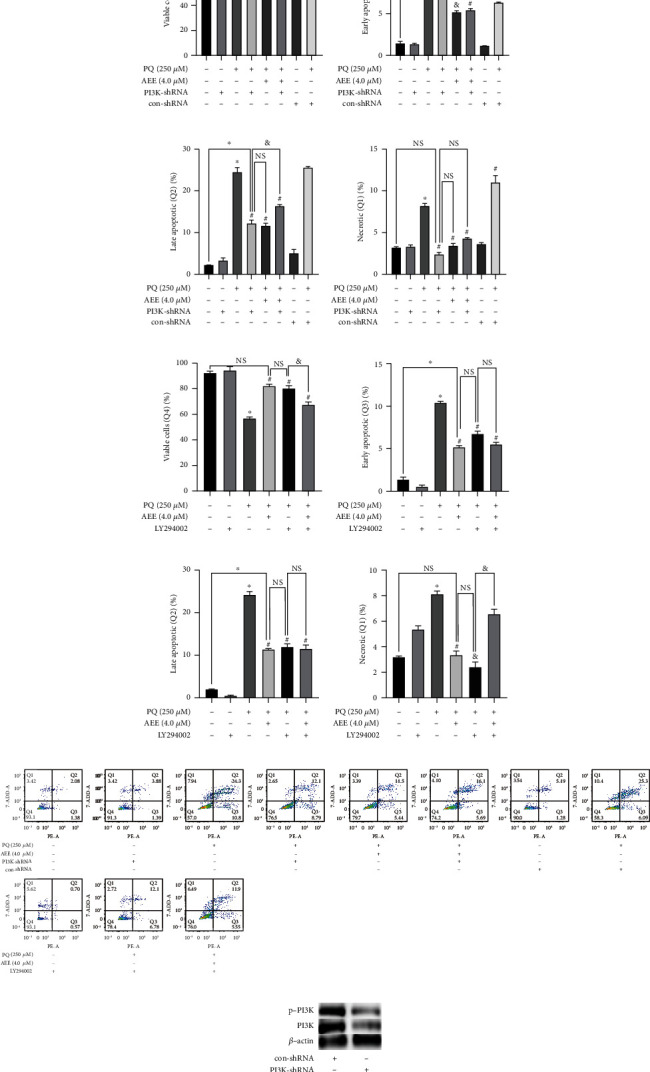
The effect of AEE on PQ-induced apoptosis after PI3K intervention with inhibitors and shRNA. (a–i) The effect of AEE on PQ-induced apoptosis after PI3K intervention with inhibitors and shRNA by flow cytometry. (j) The expression of p-PI3K and PI3K in the control-shRNA treatment groups and PI3K-shRNA treatment groups. Values are presented as the means ± SD where applicable (*n* = 3). ^∗^*p* < 0.05 compared with the control group, ^#^*p* < 0.05 compared with the PQ group, and no significant difference (NS) *p* > 0.05. “+”: with the treatments in the SH-SY5Y cells; “−”: without the treatments in the SH-SY5Y cells, ^&^*p* < 0.05 compared with the shRNA PI3K+AEE+PQ group or LY294002+AEE+PQ group.

To further explore whether the PI3K/Akt pathway is the key pathway for AEE to protect SH-SY5Y cells, PI3K inhibitors LY294002 (10 *μ*M) and shRNA were used to inhibit the expression of the PI3K/Akt signal pathway (Figure 7(g)). The results of flow cytometry showed that compared with the control group, the survival rate of SH-SY5Y cells in the PQ group was significantly reduced. PQ could increase early apoptosis (Q3), late apoptosis (Q2), and necrosis (Q1) of SH-SY5Y cells, while 4 *μ*M AEE could significantly attenuate the above phenomenon (Figures 7(a)–7(d) and 7(i)). We have reinterpreted the data for Figures 7(a)–7(d) (populations of cells Q1, Q2, Q3, and Q4).

Compared with the PQ+AEE group, there was no significant difference in cell survival rate, late apoptotic cells, and necrotic cells in the shRNA PI3K+PQ or LY294002+PQ group (Figures 7(a), 7(c), 7(d), 7(e), 7(g), 7(h), and 7(i)). Interestingly, compared with the PQ+AEE group, there were significant differences in early apoptotic cells in the shRNA PI3K+PQ group (Figure 7(b)). On the contrary, compared with the PQ+AEE group, there was no significant difference in early apoptotic cells in the LY294002+PQ group (Figure 7(f)). The results showed that the effect of inhibition of PI3K (by shRNA PI3K or LY294002) was protective in similar way, compared with the PQ+AEE group. However, the shRNA PI3K+PQ group was not statistically different from the PQ+AEE group, although there was some tendency in weakening by the effect of AEE in population of late apoptotic cells (Figure 7(c)). Compared with the shRNA control+PQ group, there was no significant difference in cell survival rate and necrosis in the shRNA PI3K+AEE+PQ group, while there were significant differences between early apoptotic cells and late apoptotic cells. It could be observed that the population of late apoptotic cells (Figure 7(c)) in the shRNA control+PQ group was in as much as the PQ group and much higher number of cells in the shRNA control+PQ than the PQ group in Figure 7(d). However, compared with the AEE+PQ group, there was no significant difference in cell survival rate, early apoptotic cells, and late apoptotic cells in the LY294002+PQ group, while there were significant differences in necrotic cells (Figures 7(e)–7(i)). From Figure 7(h), LY294002 inhibitor of PI3K seemed to reverse the effect of AEE on PQ, but it was protective when given with PQ (Q1, necrotic cells). This may result from mechanical injury in test operation because LY294002 can inhibit cell apoptosis if all apoptosis cells in Q1, Q2, and Q3 were incorporated to analyze. When LY294002 inhibitor is used, the protective effect of AEE on SH-SY5Y cells was significantly weakened, which showed there were some antagonisms between LY294002 and AEE.

**Figure 8:**
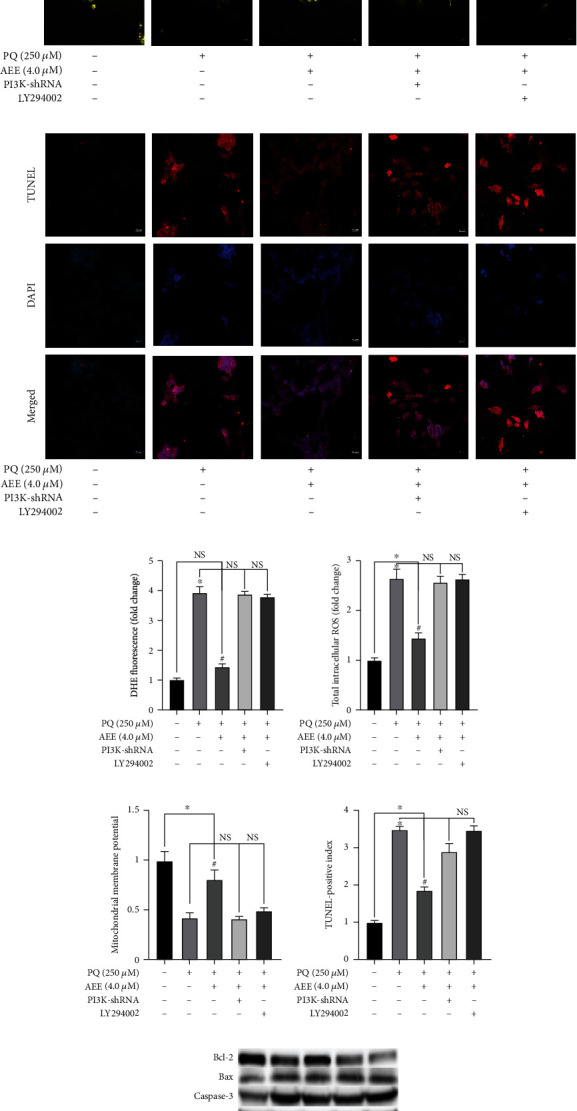
Intervention of PI3K with inhibitors and shRNA could weaken the protective effect of AEE. (a, e) Intervention of PI3K with inhibitors and shRNA could weaken the inhibitory effect of AEE on DHE. Scale bar = 50 nm. (b, f) Intervention of PI3K with inhibitors and shRNA could weaken the inhibitory effect of AEE on ROS. Scale bar = 50 nm. (c, g) Intervention of PI3K with inhibitors and shRNA could weaken the effect of AEE on the improvement of mitochondrial membrane potential. Scale bar = 20 nm. (d, h) Intervention of PI3K with inhibitors and shRNA could weaken the protective effect of AEE on cell apoptosis via DAPI and TUNEL staining. Scale bar = 20 nm. (i–k) Intervention of PI3K with inhibitors and shRNA could weaken the protective effect of AEE on cell apoptosis via western blot. Values are presented as the means ± SD where applicable (*n* = 6). ^∗^*p* < 0.05 compared with the control group, ^#^*p* < 0.05 compared with the PQ group, and no significant difference (NS) *p* > 0.05. “+”: with the treatments in the SH-SY5Y cells; “−”: without the treatments in the SH-SY5Y cells.

Compared with the PQ group, AEE pretreatment significantly inhibited PQ-induced superoxide anion, intracellular ROS, and the number of TUNEL-positive cells and significantly improved ΔΨ*m* in SH-SY5Y cells (Figures 8(a)–8(h)). Compared with the PQ group, the levels of superoxide anion, intracellular ROS, ΔΨ*m*, and the number of TUNEL-positive cells were not significantly different in the LY294002+PQ treatment group or the PI3K shRNA+PQ treatment group. Western blot results showed that the ratio of Bcl-2 and Bax and the expression of caspase-3 were not significantly different in the LY294002+PQ treatment group or the PI3K shRNA+PQ treatment group (Figures 8(i)–8(k)). The above results showed that inhibition of PI3K will weaken AEE-mediated protection.

## 4. Discussion

As we all know, the pathogenesis of PD is mediated by excessive production of ROS, which will eventually lead to the loss of mitochondrial membrane potential, resulting in the activation of caspase cascade [38–41]. In this study, PQ induced oxidative stress in SH-SY5Y cells, and the production of ROS was the initial event that mediated the death of SH-SY5Y cells. The present study revealed that AEE pretreatment significantly reduced the excessive production of intracellular ROS and mtROS, which may be one of the reasons for AEE inhibiting PQ-induced apoptosis in SH-SY5Y cells. Based on further study, cell stimulation by external factors will increase the level of Bax, and Bax promotes the release of cytochrome c from the mitochondria to the cytoplasm, thus inducing neuronal apoptosis [42–44]. PQ could significantly increase the expression of Bax, and AEE pretreatment could attenuate the expression of Bax induced by PQ in a concentration-dependent manner.

Mitochondrial dysfunction is considered a critical mechanism underlying the pathogenesis of PD. PQ promoted oxidative stress at mitochondrial level, which, in turn, impacted on the morphology of these organelles and, ultimately, on cell viability [45]. Studies showed that PQ could induce the formation of free radicals, mitochondrial dysfunction, mitosis, and activation of PINK1 protein in SY5Y cells. Lee et al. could attenuate PQ-induced neurotoxicity by mediating mitochondrial dysfunction and mitosis [46]. Ju et al. reported that PQ induces the accumulation of double-membrane autophagic vacuoles (AVs) in the cytoplasm of SH-SY5Y cells. PQ enhanced ROS-mediated neuroinflammation, oxidative stress, and apoptosis in SH-SY5Y cells. However, the effect of PQ was counteracted by vasicinone treatment, which activated the IGF-1R/AKT/PI3K signaling pathway to inhibit MAP kinases and the expression of apoptotic proteins such as Bax and Bad, inhibited cytochrome c release, and inhibited the cleavage of caspase-9, caspase-3, and PARP, suppressing cell death [47]. PQ also could induce mitochondrial dysfunction through NRF2 transcription factors and miR-34a and increase the expression of Bcl-2 family proteins and BDNF mRNA [48]. However, some studies showed that PQ-induced apoptosis of SH-SY5Y cells is independent of mitochondrial dysfunction [49]. PQ could directly participate in the oxidative cycle by increasing the level of ROS and lead to caspase-independent cell death, which is similar to programmed cell necrosis.

The important characteristics of apoptosis are caspase cascade activation, DNA fragmentation, and nuclear pyknosis [50, 51]. Bax exhibits proapoptotic actions, whereas Bcl-2 has an antiapoptotic effect [52]. The delicate balance between Bax and Bcl-2 regulates cell integrity and controls cell survival. When this balance is broken by external factors, it activates the signaling process of cell death [53]. In mammalian cells, Bcl-2 family proteins regulate the release of mitochondrial cytochrome c into the cytoplasm and further activate caspase family proteins [54, 55]. The studies showed that AEE protects nerve cells from oxidative stress and apoptosis induced by PQ, which is proved by the decrease of Bax protein level and the increase of Bcl-2 protein level.

Our previous studies have shown that AEE treatment significantly reduced H_2_O_2_-induced oxidative stress in HUVECs via mitochondria-lysosome axis and Bcl2 was an important regulation target of AEE to protect cells from oxidative stress [28]. AEE decreased lipid peroxidation and enhanced antioxidant ability in the HUVECs and mitigated mitochondrial dysfunction induced by H_2_O_2_. Similar to this study, AEE could also improve the mitochondrial dysfunction of SH-SY5Y cells induced by PQ via inhibiting oxidative stress. Different from the previous studies, AEE attenuated PQ-induced apoptosis in SH-SY5Y cells via the PI3K/AKT signal pathway.

**Figure 9:**
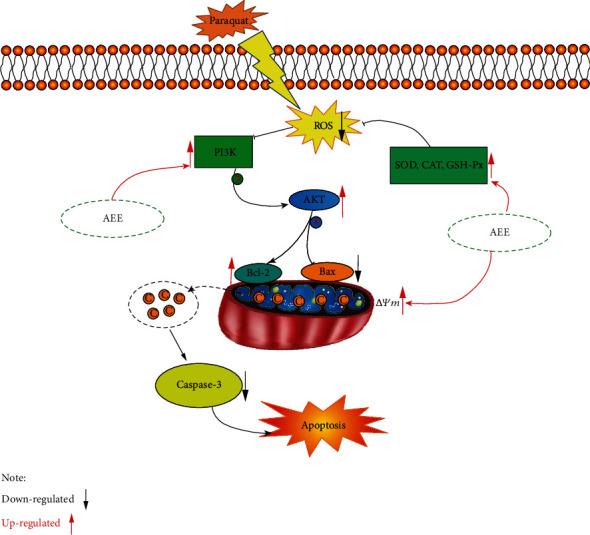
The molecular mechanism of AEE inhibiting PQ-induced apoptosis in SH-SY5Y cells.

The PI3K/Akt signaling pathway plays an important role in cell survival, differentiation, proliferation, and apoptosis [56–58]. Phosphatidylinositol 3 kinase (PI3Ks) belongs to the lipid kinase family, which phosphorylates inositol phosphate at the D-3 position of the inositol head group, resulting in the production of the D-3 phosphate. PI3K mediates extracellular signal transduction and regulates a variety of cellular events, including cell mitosis, cell survival, and membrane transport. According to the enzyme domain structure and substrate specificity of PI3K, it can be divided into three categories in mammals (I-III). Among them, the class I subfamily is the most widely studied. The class I subfamily consists of four catalytic subunits, including three IA subunits (p110-*α*, p110-*β*, and p110-*δ*) and one IB subunit (p110-*γ*). When phosphorylation of PI3K increases, it transduces signals through inositol 3-phosphate-dependent protein kinase-1 (PDK1), a serine/threonine kinase. PDK1 is recruited to the cell membrane after PI3K activation, where it phosphorylates and activates Akt, the main medium of the PI3K signal transduction pathway. Akt, a serine/threonine kinase, is pivotal in cellular metabolism, growth, and survival [59, 60]. When Akt is activated, it plays a key role in PI3K-mediated signal transduction [61–63]. The phosphorylation of AKT can increase the expression of Bcl-2 and inhibit the expression of Bax in the mitochondria. LY294002 is not only a competitive DNA-PK inhibitor but also a commonly used PI3K drug inhibitor, which acts on the ATP binding site of PI3K enzyme, thus selectively inhibiting PI3K-Akt connection. Pretreatment with LY294002 for 2 h significantly counteracted the protective effect of AEE. Consistent with this, using shRNA to knock down PI3K has a similar result (Figure 8). PQ treatment of SH-SY5Y cells resulted in excessive production of intracellular ROS. The phosphorylation of PI3K can be inhibited by excessive production of ROS. However, AEE pretreatment could inhibit the decrease of PI3K phosphorylation induced by PQ. With the recovery of mitochondrial membrane potential, the mitochondria will reduce the release of cytochrome c and inhibit the activation of caspase family. At the same time, the enzyme activities of CAT, SOD, and GSH-Px were changed by AEE pretreatment, which further eliminated the excess ROS in the SH-SY5Y cells. Both LY294002 and shRNA could inhibit the expression of PI3K. When SH-SY5Y cells were interfered by inhibitors LY294002 and shRNA, compared with the LY294002 group, the inhibitory effect of the AEE+LY294002 group on PQ-induced apoptosis of SH-SY5Y cells was weakened. Similarly, compared with the PI3K shRNA group, the inhibitory effect of the AEE+PI3K shRNA group on PQ-induced apoptosis of SH-SY5Y cells was also weakened. The results showed that AEE can alleviate PQ-induced apoptosis of SH-SY5Y cells via upregulating the expression of p-PI3K, p-Akt, and Bcl-2 and downregulating the expression of caspase-3 and Bax (Figure 9).

## 5. Conclusion

PQ enhanced the oxidative stress and apoptosis of SH-SY5Y cells mediated by ROS. AEE pretreatment inhibited cell death by activating the PI3K/Akt signal pathway, inhibiting the expression of apoptotic proteins such as Bax and Bad, and inhibiting the cleavage of caspase-3. There is reasonable evidence to support that AEE may be a new potential drug to treat neurodegenerative diseases for further *in vivo* studies.

## Data Availability

The data used to support the findings of this study are included within the article.
